# Supplementary material for: Deletion of wfs1 Impairs Oligodendrocyte Precursor Cells Dorsal Distribution and Myelination Through the wfs1-hmgcs1 Axis in Zebrafish
Source: Biology (Basel). 2026 Aug 16;15(16):1402. doi: 10.3390/biology15161402 (PMC13510151; doi:10.3390/biology15161402)
Supplement: Supplementary file 1 [file biology-15-01402-s001.zip › File S1-Transcriptome sequencing data and differentially expressed genes (DEGs) in wfs1-- zebrafish at 5 dpf/2.KEGG/wfs1KOvsWT/UP/src/dre00514.html]

dre00514


- dre:556077

- Up regulated genes

556077\_1(2.14312060824174)

- dre:556077

- Up regulated genes

556077\_1(2.14312060824174)

- dre:557675

- Up regulated genes

557675\_1(6.73009613450247)
- dre:555344

- Up regulated genes

555344(1.63564385781251)
- dre:337131

- Up regulated genes

337131(2.36410196339933)

- dre:557675

- Up regulated genes

557675\_1(6.73009613450247)
- dre:555344

- Up regulated genes

555344(1.63564385781251)
- dre:337131

- Up regulated genes

337131(2.36410196339933)

Close
